# De novo biosynthesis of pterostilbene in an *Escherichia coli* strain using a new resveratrol O-methyltransferase from *Arabidopsis*

**DOI:** 10.1186/s12934-017-0644-6

**Published:** 2017-02-15

**Authors:** Kyung Taek Heo, Sun-Young Kang, Young-Soo Hong

**Affiliations:** 1 Chemical Biology Research Center, Korea Research Institute of Bioscience and Biotechnology, 30 Yeongudanji-ro, Ochang-eup, Chungbuk, 363-883 Republic of Korea; 2 Major of Biomolecular Science, University of Science and Technology, Daejeon, Republic of Korea

**Keywords:** Pterostilbene, Resveratrol O-methyltransferase, De novo biosynthesis

## Abstract

**Background:**

Pterostilbene, a structural analog of resveratrol, has higher oral bioavailability and bioactivity than that of the parent compound; but is far less abundant in natural sources. Thus, to efficiently obtain this bioactive resveratrol analog, it is necessary to develop new bioproduction systems.

**Results:**

We identified a resveratrol O-methyltransferase (ROMT) function from a multifunctional caffeic acid O-methyltransferase (COMT) originating from *Arabidopsis*, which catalyzes the transfer of a methyl group to resveratrol resulting in pterostilbene production. In addition, we constructed a biological platform to produce pterostilbene with this ROMT gene. Pterostilbene can be synthesized from intracellular l-tyrosine, which requires the activities of four enzymes: tyrosine ammonia lyase (TAL), *p*-coumarate:CoA ligase (CCL), stilbene synthase (STS) and resveratrol O-methyltransferase (ROMT). For the efficient production of pterostilbene in *E. coli*, we used an engineered *E. coli* strain to increase the intracellular pool of l-tyrosine, which is the initial precursor of pterostilbene. Next, we tried to produce pterostilbene in the engineered *E. coli* strain using l-methionine containing media, which is used to increase the intracellular pool of S-adenosyl-l-methionine (SAM). According to this result, pterostilbene production as high as 33.6 ± 4.1 mg/L was achieved, which was about 3.6-fold higher compared with that in the parental *E. coli* strain harboring a plasmid for pterostilbene biosynthesis.

**Conclusion:**

As a potential phytonutrient, pterostilbene was successfully produced in *E. coli* from a glucose medium using a single vector system, and its production titer was also significantly increased using a l-methionine containing medium in combination with a strain that had an engineered metabolic pathway for l-tyrosine. Additionally, we provide insights into the dual functions of COMT from *A. thaliana* which was characterized as a ROMT enzyme.

**Electronic supplementary material:**

The online version of this article (doi:10.1186/s12934-017-0644-6) contains supplementary material, which is available to authorized users.

## Background

Pterostilbene (3,5-dimethoxy-4′-hydroxy-*trans*-stilbene) is a naturally derived compound found primarily in blueberries and in the heartwood of red sandalwood (*Pterocarpus santalinus*) [1]. Pterostilbene has numerous preventive and therapeutic properties for a vast range of human diseases that include neurological, cardiovascular, metabolic, and hematologic disorders [2, 3]. Further benefits of pterostilbene have been reported in preclinical trials, in which pterostilbene was shown to be a potent anticancer agent in several malignancies [3, 4]. Pterostilbene is structurally similar to resveratrol; however, the substitution of the hydroxyl with methoxy groups increases the lipophilicity of pterostilbene over resveratrol, which results in a high bioavailability [5, 6]. These differences in the pharmacokinetics might explain the higher biological activity of pterostilbene over its parental compound resveratrol [3, 7].

**Fig. 1:**
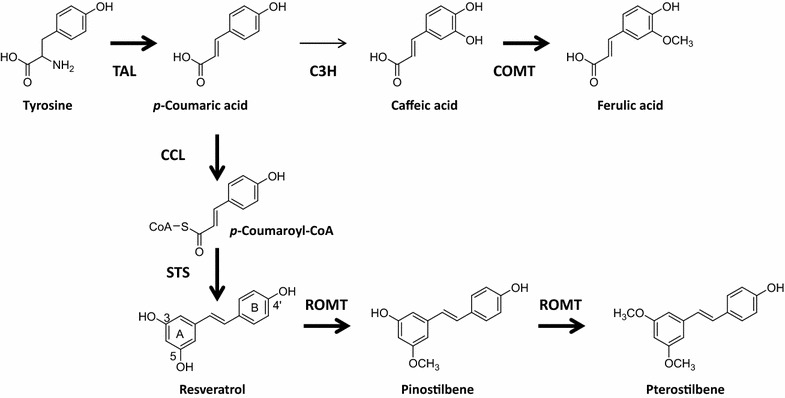
Engineered biosynthetic pathways for pterostilbene starting from l-tyrosine in *E. coli* and the proposed ferulic acid biosynthetic pathway. TAL (tyrosine ammonia-lyase), CCL (*p*-coumarate: CoA ligase), STS (stilbene synthases), ROMT (resveratrol O-methyltransferases), *p*-coumarate 3-hydroxylase (C3H), COMT (caffeic acid methyltransferase). *Bold arrows* are genes used in this study

The pterostilbene biosynthesis pathway is shown in Fig. 1. In this pathway, tyrosine is converted into *p*-coumaric acid using tyrosine ammonia lyase (TAL). The *p*-coumaric acid is then activated to *p*-coumaroyl-CoA with the *p*-coumarate CoA ligase (CCL). This *p*-coumaroyl-CoA is condensed with three molecules of malonyl-CoA via stilbene synthase (STS), which is the key enzyme in resveratrol synthesis. Resveratrol is converted into its methylated analogs, pinostilbene and pterostilbene, through resveratrol O-methyltransferase (ROMT). Schmidlin et al. [8] first reported that ROMT, which is induced by fungal infection in grapevine leaves, could catalyze the direct conversion of resveratrol into pterostilbene. However, only a few plant ROMT genes have been isolated and characterized [8–11].

Resveratrol is most commonly found in grapes and the wine made from those grapes; however, pterostilbene is found primarily in some grapes and blueberries, which is at levels of 99–520 ng/g dry berry samples compared with the highest content of 5 mg/g of resveratrol [1]. Therefore, pterostilbene has become an attractive target for bioengineering; however, only a few attempts to produce of pterostilbene have been tried in microbes and plants thus far [10–14]. Katsuyama et al. [10] reported on the production of pterostilbene in recombinant *E. coli* using the pinosylvin methyltransferase gene from *Oryza sativa*. Rimando et al. [9] reported on the accumulation of pterostilbene in tobacco and *Arabidopsis* by co-expression of O-methyltransferase and stilbene synthase. Jeong et al. [11] reported on the production of a trace amount of pterostilbene from resveratrol in *E. coli* by the expression of O-methyltransferase from *Sorghum bicolor*. Wang et al. [13] recently reported on the production of pterostilbene from *p*-coumaric acid in both *E. coli* and yeast. Meanwhile, the O-methyltransferase (OMT) family is responsible for catalyzing the transfer of a methyl group from S-adenosyl-l-methionine (SAM) to a wide range of secondary metabolites [15]. It is generally thought that some OMTs use a diverse range of substrates, including phenylpropanoids, alkaloids, and flavonoids [16, 17].

In the present study, we describe the production of pterostilbene with the recombinant *E. coli* that harbors a new artificial biosynthetic pathway. In addition, this system was achieved with the novel resveratrol O-methyltransferase (ROMT) function of COMT gene from *Arabidopsis* which was originally reported to convert caffeic acid to ferulic acid.

## Results and discussion

### Characterization of the ROMT function of the *Arabidopsis* COMT gene

**Fig. 2:**
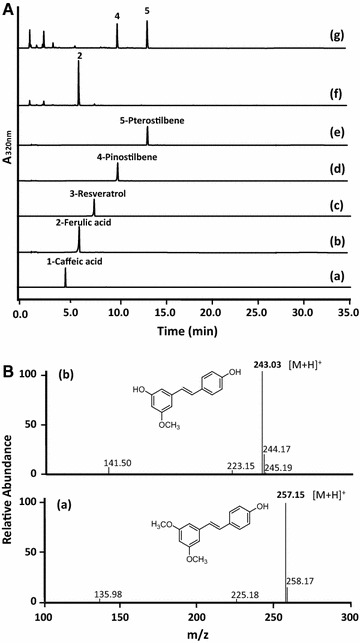
HPLC profile (**A**) and selected mass ion chromatogram (**B**) of bioconversion experiments. **A** HPLC profile of the standard caffeic acid (*a*), ferulic acid (*b*), resveratrol (*c*), pinostilbene (*d*), and pterostilbene (*e*); caffeic acid supplemented *E. coli* harboring pET-COM (C1) (*f*); resveratrol supplemented the C1 strain (*g*). The absorbance was monitored at 320 nm. **B** Selected mass ion chromatogram of (*a*) pterostilbene (*m/z* 257.15 [M + H]^+^) and (*b*) pinostilbene (*m/z* 243.03 [M + H]^+^) produced by resveratrol supplemented the C1 strain

Caffeic acid O-methyltransferase (COMT), which is originates from *Arabidopsis thaliana*, previously appears to have a wide substrate specificity [18]. To identify a new function of COMT gene from *A. thaliana*, we expressed the COMT gene in *E. coli* and screened for ROMT activity, which catalyzes the transfer of a methyl group to resveratrol resulting in methylated resveratrol, i.e., pterostilbene production. First, to monitor the ability of the *E. coli* strain to produce pterostilbene product in the presence of a resveratrol substrate, we cultured a recombinant *E. coli* strain (C1) for 60 h in the presence of 0.2 mM resveratrol and subjected the culture medium to HPLC and LC/MS to measure the level of pterostilbene (Fig. 2). The recombinant *E. coli* strain (C1) expressing the COMT gene is already known to catalyze the conversion of caffeic acid to ferulic acid (Fig. 2A). Interestingly, almost all of the resveratrol was consumed in the culture media, and pterostilbene was detected as a main peak on the HPLC profiles (Fig. 2A). The peak 5 at 13.8 min in the HPLC has the same retention time as the pterostilbene (MW = 256) standard in Fig. 2A. The major peak exhibited parent mass ion peaks at *m/z* 257.15 [M + H]^+^, which corresponded to two methylations of resveratrol (an addition of 28 Da; Fig. 2B). Also, C1 strain produced another peak 4 at 10.6 min in the HPLC, which was the same retention time as pinostilbene (MW = 242) (Fig. 2A). This peak was also accepted based on the ion peaks of *m/z* 243.03 [M + H]^+^ in the mass spectra, which corresponded to one methylation of resveratrol (an addition of 14 Da; Fig. 2B).

To determine whether the in vivo multifunctional COMT activities seen in *E. coli* are matched by the respective in vitro enzymatic activities, we expressed COMT in *E. coli* as His-tagged proteins to facilitate purification (Additional file 1: Figure S1). The substrate specificity of the purified COMT activity was investigated by measuring the conversion of substrate (6.25–400 μM caffeic acid and resveratrol) to product (ferulic acid and pinostilbene) during 30 min. To determine the *K*_*m*_ and *K*_*cat*_, we used the Lineweaver–Burk equation. As for the COMT kinetics, the *K*_*m*_ value for caffeic acid was 40.5 ± 6.6 μM and the *K*_*cat*_ was about (35.3 ± 5.7) × 10^−3^ s^−1^. The *K*_*m*_ and *K*_*cat*_ for resveratrol as a ROMT were 44.9 ± 3.2 μM and (12.8 ± 1.4) × 10^−3^ s^−1^, respectively (Table 1; Additional file 1: Figure S2). The catalytic efficiency (*K*_*cat*_/*K*_*m*_) of the COMT activity was three times higher than that of the ROMT activity. However, pinostilbene was minimally converted to pterostilbene under the same reaction conditions. To achieve higher conversion yields for pterostilbene, metabolite pattern analyses were performed for the enzyme reactions with resveratrol and pinostilbene according to reaction times of 0.5, 2, 4, 8, and 24 h, respectively (Additional file 1: Figure S3). The obtained results show that the enzyme reaction progresses to pterostilbene from resveratrol through pinostilbene. However, the initial reaction by ROMT using resveratrol proceeds more rapidly than the next reaction using pinostilbene. It is speculated that the initial reaction with resveratrol is suppressed or delayed by the accumulation of pinostilbene. Consequently, these results clearly show that this COMT from *A. thaliana* has multifunctional enzyme activity such as COMT and ROMT. Thus, COMT catalyzes the biosynthesis of pterostilbene from resveratrol, and its ROMT function is lower than that of ferulic acid synthesis, but is considerable.

**Table 1:** Kinetic analysis of the purified recombinant COMT

Substrates	*K*_*m*_ [μM]	*K*_*cat*_ [s^−1^]	Catalytic efficiency *K*_*cat*_/*K*_*m*_
Caffeic acid	40.5 ± 6.6	(35.3 ± 5.7) × 10^−3^	872.8
Resveratrol	44.9 ± 3.2	(12.8 ± 1.4) × 10^−3^	283.9

Determination of the *K*_*m*_ and *K*_*cat*_ of the COMT activity for caffeic acid to ferulic acid and ROMT activity for resveratrol to pinostilbene. Purified COMT (4 μg) was incubated with different concentrations of substrate for 30 min at 37 °C. The assay conditions are described in the “Methods” section. The data represent the mean S.D. of triplicate experiments

### Construction of a de novo artificial biosynthetic pathway in *E. coli* to produce pterostilbene

Although the production of pterostilbene has been reported by some previously bio-engineering methods [9, 11, 13, 14], we tried a de novo synthesis in *E. coli* by engineering an artificial biosynthetic pathway using newly identified ROMT. This could be a useful approach for economic production by one-pot fermentation without a precursor feeding process.

**Fig. 3:**
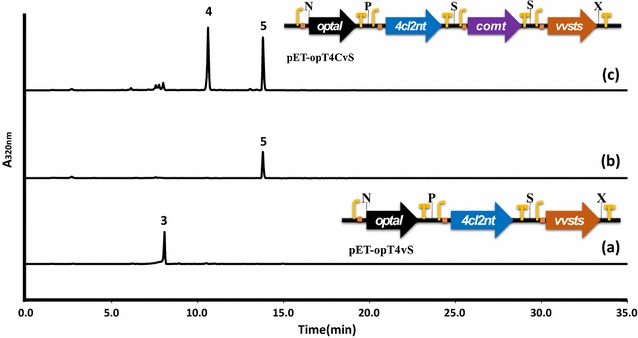
De novo biosynthesis of pterostilbene by the P1 and P2 strain. Comparison of HPLC profiles of the culture broth of P0 (*a*), P1 (*b*) and P2 (*c*) strain for 48 h. Peak 3, resveratrol; peak 4, pinostilbene; peak 5, pterostilbene. Organization of the artificial gene clusters used for the production of pterostilbene and resveratrol in *E. coli*. All constructs contained the T7 promoter, RBS in front of each gene, and T7 terminator at the rear of each gene. P, *Pac*I; S, *Spe*I; N, *Nde*I; X, *Xho*I

**Fig. 4:**
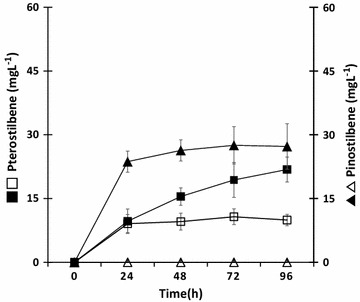
Production pattern of pterostilbene and pinostilbene for each culture time of P1 and P2 strain in M9C medium. *Error bars* indicate standard deviations of the means (n = 3). P1 strain *white*; P2 strain *black*; pterostilbene *squares*; pinostilbene *triangels*

We have previously produced resveratrol in *E. coli* harboring an artificial biosynthetic gene cluster containing the TAL, CCL, and STS genes [12, 19, 20]. Here, a plasmid that contained the artificial pterostilbene biosynthetic pathway was constructed that also included this new *comt* gene from this study. The resveratrol producing construct pET-opT4vS was used the parent vector which contains the codon-optimized TAL gene (*optal*) of *Saccharothrix espanaensis* [21], the cloned CCL gene (*4cl2nt*) of *Nicotiana tabacum* [22], and the codon-optimized STS gene (*vvsts*) of *Vitis vinifera*. This *E. coli* strain (P0) that contains the artificial biosynthetic pathway (pET-opT4vS) produces resveratrol from simple carbon sources (Fig. 3a). For the production of pterostilbene in *E. coli*, the pET-opT4CvS plasmid was constructed with additional insertion of the COMT gene (*comt*) into the parental pET-opT4vS plasmid containing the resveratrol biosynthetic pathway. The recombinant *E. coli* strain (P1), which harbors the artificial biosynthetic gene cluster (pET-opT4CvS), was cultured in a modified minimal medium (M9C). The pterostilbene peak was detected as a major peak in the culture broth of the P1 strain by HPLC (Fig. 3b). Additionally, the P1 strain was investigated using metabolite pattern analyses based on the culture times, until the production of pterostilbene was saturated after 24 h (Fig. 4). The amount of pterostilbene reached 10.7 ± 1.9 mg/L at 72 h (Table 2). This is the first report about the production of pterostilbene in a microorganism by *de novo* biosynthesis, without feeding any precursors, e.g., *p*-coumaric acid and resveratrol; therefore, further engineering of the pterostilbene biosynthetic pathway from cheap sugar sources in this microorganism could show significant economic importance.

**Table 2:** Production of pinostilbene and pterostilbene by engineered *E. coli* strains

Strain	Medium	Compound (mg/L)	
		Pinostilbene	Pterostilbene
P1	M9C	ND	10.7 ± 1.9
	M9M	ND	9.3 ± 2.8
P2	M9C	27.5 ± 4.4	19.4 ± 5.3
	M9M	17.4 ± 6.8	33.6 ± 4.1

The data were obtained after a 72 h fermentation at M9C and M9M media to the P1 and P2 strains, respectively. Each batch cultivation was done at least in triplicate, and the standard deviations are shown

*M9C* modified minimal medium containing 25 g/L CaCO_3_, *M9M* M9C medium with the addition of 1 mM l-methionine, *ND* not detected

### Improved production of pterostilbene in a l-tyrosine overproducing *E. coli* strain

To improve for the production yield, we used the pterostilbene production system in a l-tyrosine overproducing *E. coli* strain. *p*-Coumaric acid is the pivotal intermediate of the resveratrol and pterostilbene pathway starting from the deamination of tyrosine or the hydroxylation of cinnamic acid. Our strategy to harness microorganisms for the production of pterostilbene was to design and express artificial pathways with bacterial TAL. Thus, l-tyrosine serves as an initial endogenous precursor to the pterostilbene biosynthesis pathway. Recently, we reported on an engineered l-tyrosine overproducing *E. coli* ΔCOS1 strain through deregulation of the aromatic amino acid biosynthesis pathway [23]. The genome engineered l-tyrosine producer, *E. coli* ΔCOS1, showed a substantial capacity for *p*-coumaric acid production through *tal* gene expression [23]. Therefore, it is a suitable platform strain for the production of resveratrol and pterostilbene, using l-tyrosine as a common precursor.

Using the same experimental conditions described above, the tyrosine-overproducing *E. coli* ΔCOS1 strain (P2) harboring the pET-opT4CvS vector produced 19.4 ± 5.3 mg/L of pterostilbene after 72 h culture (Table 2). This productivity shows 1.8-fold improvement over the titers of the original producer (P1). At the same time, a significant amount of accumulated pinostilbene (27.5 ± 4.4 mg/L) was also detected, and there was trace accumulation of the intermediate *p*-coumaric acid and resveratrol (Fig. 3c). The amount of accumulated pinostilbene was slightly reduced and pterostilbene increased after 96 h culture (Fig. 4). This result means that the extra pinostilbene are not well converted to pterostilbene and accumulated in the cell. Therefore, methylation in the metabolic flow of resveratrol to pterostilbene may act as a bottleneck because of a shortage of methyl donors in the l-tyrosine overproducing cells.

### Effects of l-methionine addition on pterostilbene production

As previously stated, this COMT enzyme uses SAM as the methyl donor. SAM production is improved when supplemented with excessive l-methionine in a l-methionine S-adenosyltransferase (MAT) overexpressing strain [24]. In addition, the production of methylated compounds is remarkably improved by the feeding of SAM and l-methionine [22, 24].

**Fig. 5:**
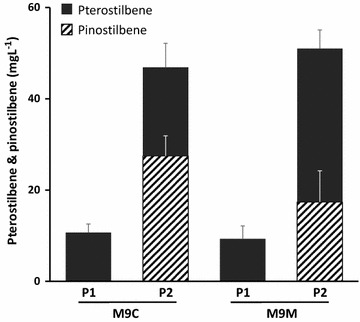
Effects of l-methionine addition on pterostilbene production. The data were obtained after a 72 h fermentation at M9C and M9M media to the P1 and P2 strains, respectively. *Error bars* show one standard deviation from triplicate experiments. The production of pinostilbene (*hatched*) and pterostilbene (*black*) in the P1 and P2 strains, respectively. The production of pterostilbene were compared with single-factor ANOVA (P < 0.05) using Microsoft Excel (P = 1.05E−28)

To investigate the acceleration of the metabolic flux to pterostilbene through the feeding of l-methionine, we supplied a final concentration of 1 mM l-methionine to the culture medium of the P1 and P2 strains, respectively. The P2 strain had a twofold higher production yield of pterostilbene (33.6 ± 4.1 mg/L) in the culture system with the additional l-methionine medium (M9M) compared with the original medium (M9C medium, 19.4 ± 5.3 mg/L) after 72 h culture (Table 2; Additional file 1: Figure S4). The production levels of pterostilbene from the tyrosine overproducing P2 strain had improvements of 1.8- and 3.6-fold over the P1 culture in M9C and M9M, respectively (Fig. 5). However, a significant amount of pinostilbene (17.4 ± 6.8 mg/L) was accumulated, indicating that it was not optimized for pterostilbene production. Therefore, the best metabolic engineered strains for pterostilbene production would need to additionally be optimized for reducing pinostilbene accumulation and quickly converting to pterostilbene. Together with these efforts, a new attempt to search for an enzyme which is better suited to perform the final methylation step from pinostilbene to pterostilbene is needed, so pterostilbene can be produced at higher concentration at a competitive level.

## Conclusions

In this study, we successfully demonstrated the de novo synthesis of pterostilbene, a di-methylated resveratrol, which is a more stable compound in vivo and potentially has more effective biological activities than that of resveratrol. This system was achieved with the novel resveratrol O-methyltransferase (ROMT) function of COMT gene from *A. thaliana* which originally was reported to convert caffeic acid to ferulic acid. The purified recombinant COMT showed significant ROMT activity catalyzing the conversion of resveratrol to pterostilbene. Furthermore, the de novo production of pterostilbene from a tyrosine overproducing strain (P2) with the addition of l-methionine to the medium was determined to be 3.6-fold over the *E. coli* P1 strain. The titers of the pterostilbene reached up to 33.6 ± 4.1 mg/L after 72 h of culturing in a minimal medium containing 1 mM l-methionine.

## Methods

### Bacterial strains, plasmids, and chemicals

The strains and plasmids used in this study are listed in Additional file 1: Table S1. Antibiotics were added to the medium as required at the following concentrations: ampicillin, 100 mg/L; kanamycin, 50 mg/L. T-blunt vector (Solgent, Korea) was used in the polymerase chain reaction (PCR) cloning. pET-22b(+) and pET-28a(+) were purchased from Novagen (USA). Caffeic acid, ferulic acid, resveratrol, and pterostilbene were purchased from Sigma-Aldrich (USA), pinostilbene was purchased from Tokyo Chemical Industry, Co (Japan) as a standard for compound identification by HPLC.

### DNA manipulation

The restriction enzymes (NEB, USA; Takara, japan), KOD-plus-DNA polymerase (TOYOBO, japan), an AccuPower Ligation kit (Bioneer, Korea) and DNA ligation kit (Takara, japan) were used according to the manufacturers’ instructions. The optimized tyrosine ammonia lyase gene (*optal*) from *Saccharothrix espanaensis* [GenBank: DQ357071], cinnamate/*p*-coumarate: CoA ligase gene (*4cl2nt*) from *Nicotiana tabacum* [GenBank: AAB18638], stilbene synthase gene (*vvsts*) from *Vitis vinifera* [GenBank: NM_001281005.1] and caffeic acid-O-methyltransferase gene (*comt*) from *Arabidopsis thaliana* [GenBank: AY062837.1; *Arabidopsis* gene number: AT5G54160.1] were synthesized.

### Enzyme purification

After cultivation of *E. coli* BL21(DE3) harboring the pET-COM [20], cells were harvested by centrifugation at 4000 rpm for 20 min at 4 °C. Harvested cells were re-suspended in lysis buffer (50 mM Tris–HCl, 300 mM NaCl, 10 mM imidazole, pH 7.4), and disrupted by sonication while chilled on ice for 30 min. Cell lysates were centrifuged at 15,000 rpm for 10 min at 4 °C, and the soluble fractions were collected from the supernatant. Soluble fractions were mixed with the His-Hyper Agarose resin (Lugen Sci. Co. Korea.) in poly-prep chromatography columns (Bio-Rad). After the binding of the enzyme, which was tagged with polyhistidine, the resin was washed with 50 mL of wash buffer (equilibration buffer containing 50 mM imidazole), and enzymes were eluted using 6 mL of elution buffer (equilibration buffer containing 250 mM imidazole). Protein samples were analyzed using 10% (w/v) SDS–polyacrylamide gel electrophoresis (SDS–PAGE). After gel electrophoresis, gels were stained with the gel staining solution (LPS solution, Korea).

### Quantification of enzyme activity

The purified recombinant COMT protein were incubated in a total volume of 100 μL of 100 mM potassium phosphate buffer (pH 7.8) containing 0.5 mM S-adenosyl-l-methionine, 4 μg COMT protein and 6.25–400 μM substrate at 37 °C for 30 min. After reaction, 20 μL aliquot was subjected to HPLC as described above. The protein concentration was determined by the Bradford method using a protein assay dye (Bio-Rad, Hercules, CA, USA). The methylation activity of COMT were examined using caffeic acid and resveratrol to ferulic acid and pinostilbene, respectively. The substrate affinity (*K*_*m*_) and turnover number (*K*_*cat*_) values were calculated from Lineweaver–Burk plots. The analysis was performed in triplicate.

### Construction of pterostilbene expression vector

The four genes (*optal*, *4cl2nt, comt* and *vvsts*) were independently cloned into pET-28a(+) vectors [19–21, 23]. Using the *optal*, *4cl2nt*, *comt*, and *vvsts* genes as templates, four DNA fragments were amplified by PCR with the appropriate pairs of primers. In order to assemble the pET-opT4CvS vector, the TAL coding region was amplified using pET-opTAL as a template with the primer opTAL-F (5′-CAT ATGACCCAGGTGGTTGAACGCC-3′) and Cpac (the sequence is located downstream of the T7 terminator region of the pET vector and contains the designed *Pac*I site: 5′-TTAATTAATGCGCCGCTACAGGGCGCGTCC-3′), the CCL coding region was amplified using pET-4cl2nt as a template with the primer Npac (the sequence is located upstream of the T7 promoter region of the pET vector and contains the designed *Pac*I site: 5′-TTAATTAATCGCCGCGACAATTTGCGACGG-3′) and Cspe (the sequence is located downstream of the T7 terminator region of the pET vector and the sequence contains the designed *Spe*I site 5′-ACTAGT TCCTCCTTTCAGCAAAAAACCCCTC-3′), the STS coding region was amplified using pET-STS as a template with the primer Nspe (the sequence is located upstream of the T7 promoter region of the pET vector and contains the designed *Spe*I site 5′-ACTAGTAGGTTGAGGCCGTTGAGCACCGCC-3′) and STS-R(5′-CTCGAGTTAGTTG GTGACCATCGGG-3′). Each of the amplified fragments were digested with corresponding sites and cloned between the *Nde*I and *Xho*I digested pET-28a(+) via ligation, which resulted in pET-opT4vS. The COMT coding region was amplified using pET-COM as a template with the primer Nspe and Cspe. A 1.6 kb *Spe*I fragment containing the *comt* gene was cloned between the *Spe*I digested pET-opT4vS which resulted in pET-opT4CvS (Fig. 3).

### Culture conditions for production

Recombinant *E. coli* strains harboring plasmids were grown at 37 °C in a Luria–Bertani (LB) medium containing 50 μg/mL kanamycin. The overnight culture was inoculated into fresh LB medium supplemented with the same concentration of kanamycin. The culture was grown at 37 °C to an optical density at 600 nm (OD_600_) of 0.6, and IPTG was added to the final concentration of 1 mM, and the culture was incubated for 6 h at 26 °C. The cells were harvested by centrifugation, suspended, and incubated at 26 °C in a modified M9 minimal medium (M9C; Na_2_HPO_4_·7H_2_O 12.8 g/L, KH_2_PO_4_ 3 g/L, NaCl 0.5 g/L, NH_4_Cl_2_ 1 g/L, yeast extract 0.25 g/L, 2 mM MgSO_4_, 0.1 mM CaCl_2_, CaCO_3_ 25 g/L, Glucose 15 g/L, 1 mM IPTG and appropriate antibiotics) and addition 1 mM l-methionine to M9C medium (M9M). For the feeding experiments, the cultures were supplemented with caffeic acid and resveratrol (final concentration: 0.2 mM), respectively, and allowed to grow for an additional 60 h.

### Detection and quantification of the products

Ten milliliters of culture was extracted with an equal volume of ethyl acetate. The ethyl acetate was dried and resuspended in 800 μL of methanol. Twenty microliters of the extract was applied to a J’sphere ODS-H80 column (4.6 × 150 mm i.d., 5 μm; YMC, Japan) using a high performance liquid chromatography (HPLC) system [CH_3_CN–H_2_O (0.05% trifluoroacetic acid) 20–100% acetonitrile (CH_3_CN) for 20 min, 100% CH_3_CN for 5 min, at flow rate of 1 mL/min; Thermo Scientific, USA] equipped with a photodiode array detector. Samples was dissolved in a methanol and analyzed by electrospray ionization (ESI) mass spectrometry in the positive ion mode using a Thermo U3000-LTQ XL (Thermo Scientific, USA) system coupled to the ion trap mass spectrometer with a ESI (Electrospray ionization) source operating in the positive mode. Two microliters of the sample was injected into a HSS T3 C18 column (2.1 × 150 mm; 2.5 μm particle size) from Waters; the mobile phase used for gradient elution consisted of CH_3_CN (0.1% formic acid) as system A and H_2_O (0.1% formic acid) as system B. The flow rate was 0.3 mL/min. The gradient elution program started with 5% A, raised A to 100% in the following 15 min, remained at 100% A for 5 min, and returned to the initial condition (5% A) within the following 5 min. The data-dependent mass spectrometry experiments were controlled using the menu driven software provide with the Xcalibur system (version 2.2 SP1.48; Thermo Scientific). Quantification of the three above-mentioned compounds was based on the peak areas of absorbance at 320 nm. For the quantification of ferulic acid, pinostilbene, and pterostilbene, the HPLC analysis was followed as our previously described methods [12, 21]. The data shown in this study were generated from triplicate independent experiments.

## Additional file

**Additional file 1.** Additional figures and tables.
