# Supplementary material for: De novo biosynthesis of pterostilbene in an Escherichia coli strain using a new resveratrol O-methyltransferase from Arabidopsis
Source: Microb Cell Fact. 2017 Feb 15;16:30. doi: 10.1186/s12934-017-0644-6 (PMC5312575; doi:10.1186/s12934-017-0644-6)
Supplement: Supplementary file 1 — Additional file 1. Additional figures and tables. [file 12934_2017_644_MOESM1_ESM.pdf]

Supporting data

De novo* biosynthesis of pterostilbene in an *Escherichia coli* strain using a new resveratrol O-methyltransferase from *Arabidopsis

Kyung Taek Heo [1,2*], Sun-Young Kang [1*], Young-Soo Hong [1,2,§]

Legends for Table and Figures

Table S1. Plasmids and strains used in this study

Figure S1. Purification of N-terminal His 6-tagged COMT. Protein samples were separated by SDS-PAGE and stained with Coomassie blue. M, molecular mass standards; lane 1, total proteins in 20-uL aliquots of bacterial culture without IPTG; lane 2, total proteins in 20-uL aliquots of bacterial culture with IPTG; lane 3 & 4, 60 μg and 20 μg of purified COMT by affinity chromatography, respectively.

Figure S2. Lineweaver–Burk plots. Determination of the K_m and K_{cat} of COMT activity for caffeic acid to ferulic acid (A) and ROMT activity for resveratrol to pinostilbene (B). Purified COMT (4 μg) was incubated with different concentrations of substrate for 30 min at 37°C. The K_m and K_{cat} values were determined using Lineweaver–Burk plots. The data represent the mean S.D. of triplicate experiments.

Figure S3. HPLC time-course profiles for the ROMT-catalyzed conversion of resveratrol (A) and pinostilbene (B) to pterostilbene. Peak 1, resveratrol; peak 2, pinostilbene; peak 3, pterostilbene.

Figure S4. Production pattern of pterostilbene and pinostilbene for each culture time of P1 (white) and P2 (black) strain in M9M medium, repectively. Error bars indicate standard deviations of the means (n=3). Pterostilbene, squares; pinostilbene, triangels.

References

1. Kang SY, Choi O, Lee JK, Hwang BY, Uhm TB, Hong YS: **Artificial biosynthesis of phenylpropanoic acids in a tyrosine overproducing *Escherichia coli* strain.** *Microb Cell Fact* 2012, **11:**153.

2. Choi O, Wu CZ, Kang SY, Ahn JS, Uhm TB, Hong YS: **Biosynthesis of plant-specific phenylpropanoids by construction of an artificial biosynthetic pathway in *Escherichia coli*.** *J Ind Microbiol Biotechnol* 2011, **38:**1657-1665.

3. Kang SY, Lee JK, Jang JH, Hwang BY, Hong YS: **Production of phenylacetyl-homoserine lactone analogs by artificial biosynthetic pathway in *Escherichia coli*.** *Microb Cell Fact* 2015, **14:**191.

4. Miroux B, Walker JE: **Over-production of proteins in *Escherichia coli*: mutant hosts that allow synthesis of some membrane proteins and globular proteins at high levels.** *J Mol Biol* 1996, 260:289-298

5. Kang SY, Choi O, Lee JK, Ahn JO, Ahn JS, Hwang BY, Hong YS: **Artificial *de novo* biosynthesis of hydroxystyrene derivatives in a tyrosine overproducing *Escherichia coli* strain.** *Microb Cell Fact* 2015, **14:**78.

Table S1. Plasmids and strains used in this study

Plasmid or strain	Relevant Characteristics	Source
**Plasmid**		
pET-28a(+)	f1 ori, T7 promoter, Kan^R	Novagen
pET-opTAL	pET-28a(+) carrying codon-optimized TAL gene (*optal*) from *Saccharothrix espanaensis*	Kang. *et al*. [1]
pET-COM	pET-28a(+) carrying COMT gene (*com*) from *Arabidopsis thaliana*	Choi *et al*. [2]
pET-4CL2nt	pET-28a(+) carrying codon-optimized CCL gene (*4cl2nt*) from *Nicotiana tabacum*	Kang. *et al*. [3]
pET-vvSTS	pET-28a(+) carrying codon-optimized STS gene (*vvsts*) from *Vitis vinifera*	This study
pET-opT4vS	pET-28a(+) carrying *optal*, *4cl2n*t and *vvsts* genes	This study
pET-opT4CvS	pET-28a(+) carrying *optal*, *4cl2n*t, *com* and *vvsts* genes	This study
**Strain**		
E. coli C41(DE3)	derivative strain of *E. coli* BL21(DE3)	Miroux B & Walker JE [4]
ΔCOS1	*E. coli* C41(DE3); Δ*tyrR*::*tyrA*^{fbr}, *aroG*^{fbr}; tyrosine overproducing strain	Kang. *et al*. [5]
C1	*E. coli* C41(DE3) harboring pET-COM	This study
P0	*E. coli* C41(DE3) harboring pET-opT4vS; resveratrol producer	This study
P1	*E. coli* C41(DE3) harboring pET-opT4CvS; pterostilbene producer	This study
P2	*E. coli* ΔCOS1 harboring pET-opT4CvS; pterostilbene producer	This study

Figure S1.

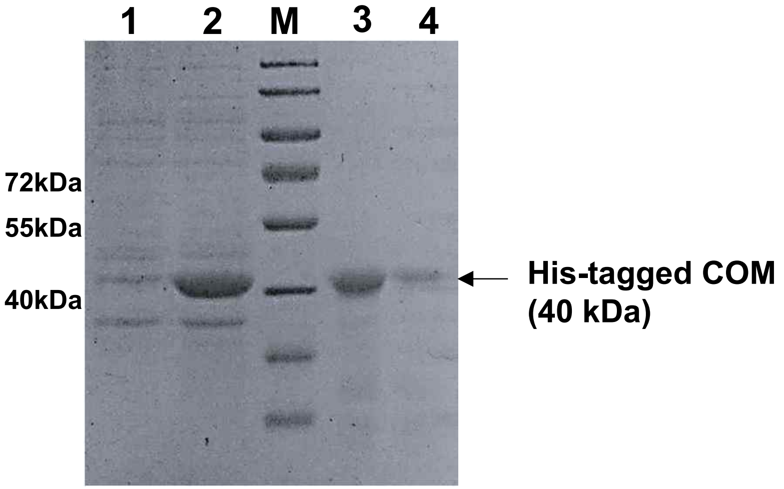

Figure S2.

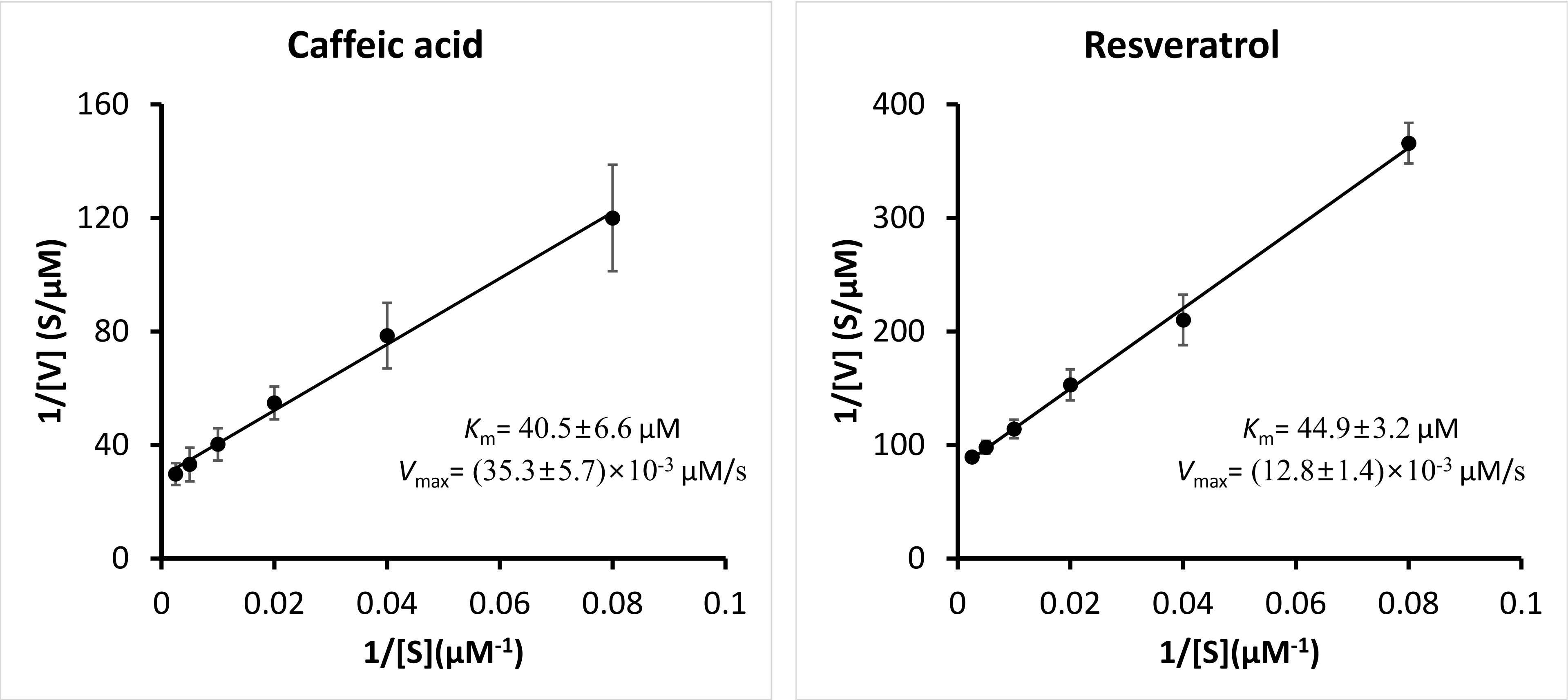
Caffeic acid
160
120
80
40
0
1/[V] (S/μM)
0
0.02
0.04
0.06
0.08
0.1
1/[S](μM-1)
Km= 40.5±6.6 μM
Vmax= (35.3±5.7)×10-3 μM/s
Resveratrol
400
300
200
100
0
1/[V] (S/μM)
0
0.02
0.04
0.06
0.08
0.1
1/[S](μM-1)
Km= 44.9±3.2 μM
Vmax= (12.8±1.4)×10-3 μM/s

Figure S3.

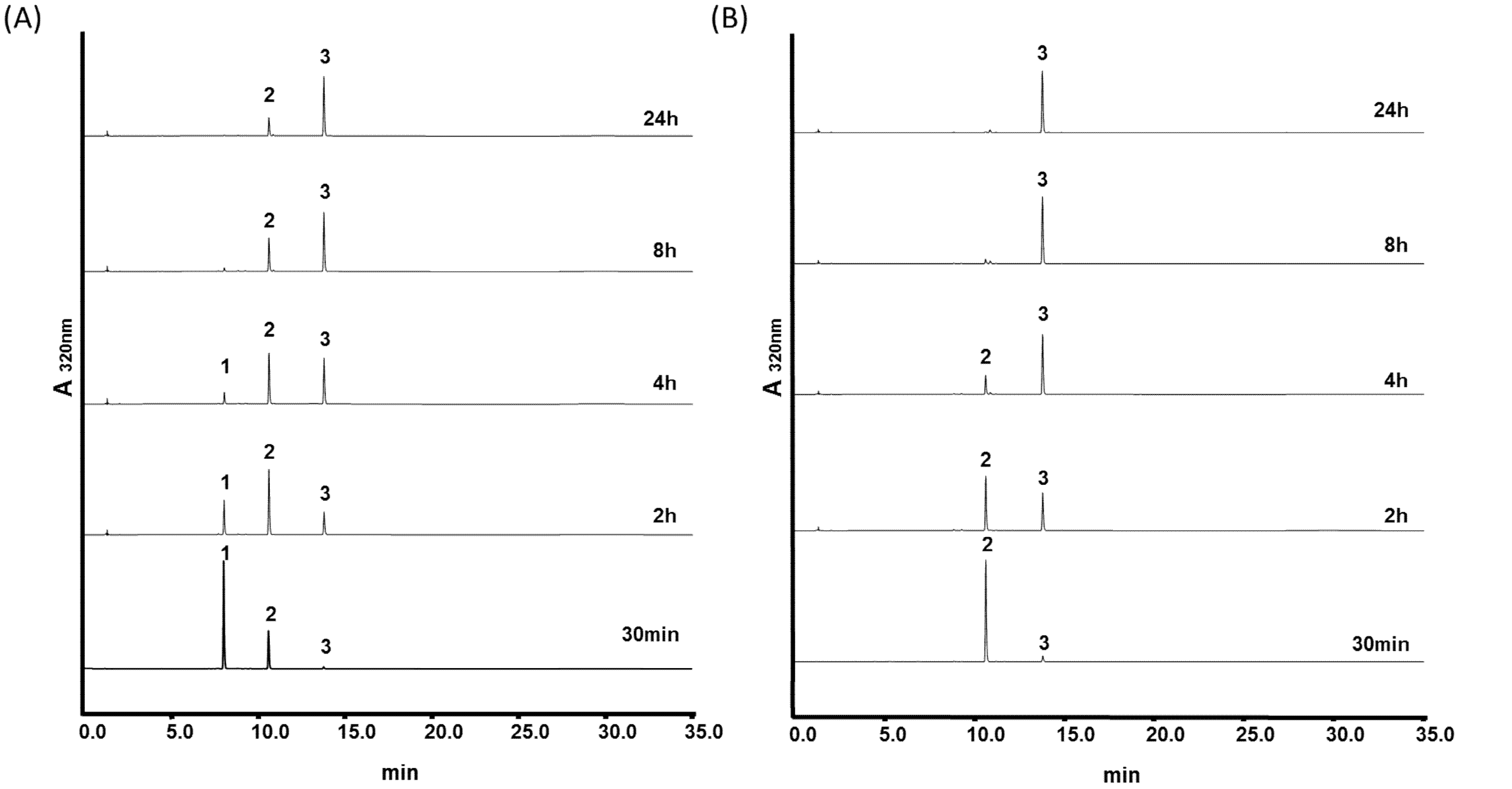

(A)
(B)
A 320nm
24h
8h
4h
2h
30min
1
2
3
0.0
5.0
10.0
15.0
20.0
25.0
30.0
35.0
min

Figure S4

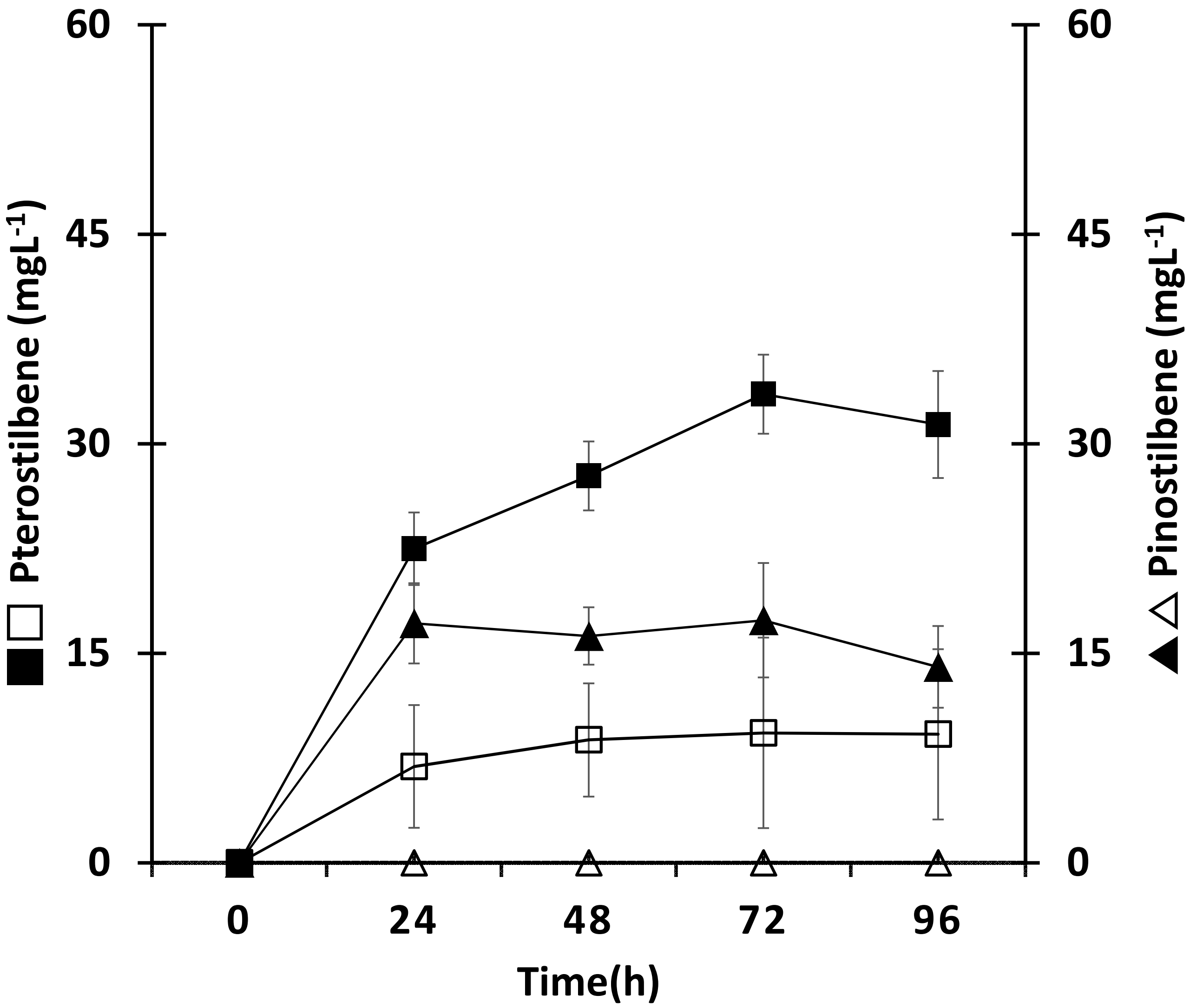

60
45
30
15
0
■ □ Pterostilbene (mgL-1)
▲ △ Pinostilbene (mgL-1)
0
24
48
72
96
Time(h)
